# Influence of the π-coordinated arene on the anticancer activity of ruthenium(II) carbohydrate organometallic complexes

**DOI:** 10.3389/fchem.2013.00027

**Published:** 2013-10-31

**Authors:** Muhammad Hanif, Samuel M. Meier, Alexey A. Nazarov, Julie Risse, Anton Legin, Angela Casini, Michael A. Jakupec, Bernhard K. Keppler, Christian G. Hartinger

**Affiliations:** ^1^School of Chemical Science, The University of AucklandAuckland, New Zealand; ^2^Institute of Inorganic Chemistry, University of ViennaVienna, Austria; ^3^Department of Chemistry, COMSATS Institute of Information TechnologyAbbottabad, Pakistan; ^4^Research Platform “Translational Cancer Therapy Research”, University of ViennaVienna, Austria; ^5^Department of Chemistry, Moscow State UniversityMoscow, Russia; ^6^Institut des Sciences et Ingénierie Chimiques, Ecole Polytechnique Fédérale de LausanneLausanne, Switzerland; ^7^Pharmacokinetics, Toxicology and Targeting, Research Institute of Pharmacy, University of GroningenGroningen, Netherlands

**Keywords:** anticancer activity, bioorganometallic chemistry, carbohydrates, phosphorus ligands, ruthenium arene compounds

## Abstract

The synthesis and *in vitro* cytotoxicity of a series of Ru^II^(arene) complexes with carbohydrate-derived phosphite ligands and various arene co-ligands is described. The arene ligand has a strong influence on the *in vitro* anticancer activity of this series of compounds, which correlates fairly well with cellular accumulation. The most lipophilic compound bearing a biphenyl moiety and a cyclohexylidene-protected carbohydrate is the most cytotoxic with unprecedented IC_50_ values for the compound class in three human cancer cell lines. This compound shows reactivity to the DNA model nucleobase 9-ethylguanine, but does not alter the secondary structure of plasmid DNA, indicating that other biological targets are responsible for its cytotoxic effect.

## Introduction

**Figure 1:**
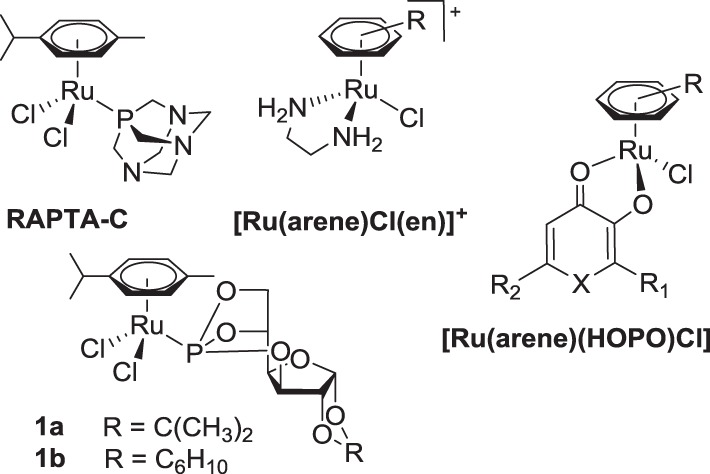
**Structures of selected Ru^II^(arene) anticancer complexes**.

In recent years, half-sandwich Ru^II^(arene) complexes (Figure 1) have been shown to have promising anticancer activity that is in some cases comparable or superior to that of established anticancer drugs (Dyson, 2007; Peacock and Sadler, 2008; Hartinger and Dyson, 2009; Gasser et al., 2011; Hartinger et al., 2012). The best investigated examples of this class are [Ru^II^(arene)(pta)Cl_2_] (RAPTA; pta = 1,3,5-triaza-7-phosphatricyclo-[3.3.1.1]decane) (Dyson, 2007), [Ru^II^(arene)(en)Cl]^+^ (en = 1,2-diaminoethane) (Peacock and Sadler, 2008) and [Ru^II^(arene)(HOPO)Cl] complexes (HOPO = hydroxypyr(id)one) (Kandioller et al., 2011). The arene ligand plays a major role in the anticancer activity of ethylene-1,2-diamine compounds and related drug candidates (Habtemariam et al., 2006; Hanif et al., 2010b), while in other cases it has less impact on cytotoxicity (Mendoza-Ferri et al., 2009), unless modified with bioactive groups (Hartinger et al., 2012). Furthermore, the arene ligand imparts hydrophobic character to the molecule, which facilitates the passive diffusion through the cell membrane, enhancing the cellular accumulation. Some arene ligands facilitate the interaction of Ru(arene) complexes with nucleobases (Chen et al., 2003) and proteins (Casini et al., 2008). However, in general the co-ligands at the metal center determine the anticancer activity of this compound class. For example, the combination of ligands en/Cl yields a complex with activity in a xenograft model (Aird et al., 2002) while pta derivatives reduce the number and weight of metastases (Dyson, 2007).

In recent years, we have reported the development of phosphorus-substituted sugar derivatives with the aim to exploit the enhanced glucose uptake in tumors due to increased glycolytic activity in cancer cells (Berger et al., 2008; Hanif et al., 2010a, 2011, 2012a,b; Nazarov et al., 2012). These organometallic compounds exhibit selective cytotoxicity in tumorigenic cell lines, and dichlorido(η^6^-*p*-cymene)(3,5,6-bicyclophosphite-1,2-*O*-cyclohexylidene-α-D-glucofuranoside)ruthenium(II) **1b** was found to be more cytotoxic than RAPTA-C in *in vitro* assays (Berger et al., 2008). The carbohydrate compounds are prone to hydrolysis, and they undergo aquation of the first halido ligand in aqueous solution, followed by hydrolysis of a P–O bond of the phosphite ligand, and finally formation of dinuclear species (Berger et al., 2008). The aquation can be hindered or suppressed by replacing ruthenium with osmium and chlorido ligands with dicarboxylates (Hanif et al., 2010a, 2011).

Functionalizing metal-arene compounds with modified sugars should yield hybrid molecules with altered pharmacological properties, such as improved biocompatibility, bioavailability, activity and targeting potential (Gottschaldt and Schubert, 2009). Furthermore, the biophysical characteristics of compounds are significantly modified by attaching carbohydrate moieties to metal-arene units (Hartinger et al., 2008). In order to study the effect of the arene ligand on anticancer potency, a series of new Ru^II^(arene) complexes bearing carbohydrate-derived phosphite ligands was prepared and compared to structurally related RAPTA derivatives, also with regard to their cathepsin B inhibitory activity, which is a potential target for anticancer metallodrugs (Fricker, 2010).

## Materials and methods

### Materials

All chemicals were obtained from commercial suppliers and used as received and were of analytical grade except for methanol and CH_2_Cl_2_ that were dried using standard procedures. The complexes bis[dichlorido(η^6^-biphenyl)ruthenium(II)] (Mendoza-Ferri et al., 2009), bis[dichlorido(η^6^-*p*-cymene)ruthenium(II)], bis[dichlorido(η^6^-benzene)ruthenium(II)], bis[dichlorido(η^6^-toluene)ruthenium(II)] (Bennett and Smith, 1974; Bennett et al., 1982), dichlorido(η^6^-p-cymene)(3,5,6-bicyclophosphite-1,2-*O*-isopropylidene-α-D-glucofuranose)ruthenium(II) **1a**, dichlorido(η^6^-p-cymene)(3,5,6-bicyclophosphite-1,2-*O*-cyclohexylidene-α-D-glucofuranose)ruthenium(II) **1 b** (Berger et al., 2008), and the ligands 3,5,6-bicyclophosphite-1,2-*O*-isopropylidene-α-D-glucofuranoside **I** and 3,5,6-bicyclophosphite-1,2-*O*-cyclohexylidene-α-D-glucofuranoside **II** (Kochetkov et al., 1976) were synthesized using literature procedures. All reactions were carried out in dry solvents under an inert atmosphere. ^1^H, ^13^C{^1^H} and ^31^P{^1^H} NMR spectra were recorded at 25°C on a Bruker FT NMR spectrometer Avance III 500 MHz at 500.10 (^1^H), 125.75 (^13^C{^1^H}) and 202.44 MHz (^31^P{^1^H}) or a Bruker Avance 400 FT NMR spectrometer at 400.13 (^1^H), 100.63 (^13^C{^1^H}) and 161.98 MHz (^31^P{^1^H}). 2D NMR spectra were collected in a gradient-enhanced mode. Melting points were measured on a Büchi B-540 apparatus and are uncorrected. Elemental analysis was performed by the Laboratory for Elemental Analysis, Faculty of Chemistry, University of Vienna, on a Perkin–Elmer 2400 CHN Elemental Analyzer and at the Microanalytical Laboratory of the EPFL. Electrospray ionization mass spectra were recorded on a Bruker esquire_3000_. Na_2_EDTA·(p.a., Fisher Scientific), NaOH (Fluka), tris(hydroxymethyl)aminoethane, glacial acetic acid (p.a., Acros), and MilliQ H_2_O (18.2 MΩ, Synergy 185 UV Ultrapure, Millipore, France) were used for the preparation of TAE buffer for gel electrophoresis studies. Loading buffer (6×), pBR322 DNA (0.5 μg/μL) and GeneRuler™ 1 kb DNA ladder (0.5 μg/μL) were obtained from Fermentas.

#### General procedure for the synthesis of 2–6

A mixture of bis[dichlorido(η^6^-arene)ruthenium(II)] (1 eq.) and the phosphite ligand (2 eq.) in dry CH_2_Cl_2_ (30 ml) was stirred for 3 h at 40°C. The solvent was reduced to about 3 mL on a rotary evaporator, and diethyl ether (20 mL) was added resulting in orange or brown precipitates which were filtered, washed with diethyl ether (2 × 5 mL), and dried under vacuum.

#### Dichlorido(η^6^-benzene)(3,5,6-bicyclophosphite-1,2-O-cyclohexylidene-α-D-glucofuranose)ruthenium(II) 2

The title compound was synthesized from 3,5,6-bicyclophosphite-1,2-*O*-cyclohexylidene-α-D-glucofuranose (58 mg, 0.2 mmol) and [(η^6^-benzene)RuCl(μ-Cl)]_2_ (50 mg, 0.1 mmol) following the general procedure.

Yield: 104 mg (96%); m.p. 295–297°C (decomp); elemental analysis calcd. for C_18_H_23_Cl_2_O_6_PRu·0.5CH_2_Cl_2_: C 38.25, H 4.16; found: C 38.21, H 4.19%; MS (ESI^+^): *m/z*: 560.7 [M + Na]^+^; ^1^H NMR (500.10 MHz, CDCl_3_, 25°C): δ = 6.20 (brs, 1 H; H-1), 5.89 (s, 6 H; H-Ar), 5.10–5.13 (m, 1 H; H-5), 4.83 (brs, 1 H; H-3), 4.75 (brs, 1 H; H-2), 4.48-4.50 (m, 1 H; H-6), 4.31–4.33 (m, 2 H; H-6′, H-4), 1.57–1.70 (m, 10 H; C_6_H_10_) ppm. ^13^C{^1^H} NMR (125.75 MHz, CDCl_3_, 25°C): δ = 113.3 (C_*cyc*_), 105.4 (C-1), 91.2 (C-Ar), 83.4 (C-2), 79.5 (C-3), 75.7 (C-4), 75.2 (C-5), 71.5 (C-6), 36.5 (C_6_H_10_), 35.8 (C_6_H_10_), 24.8 (C_6_H_10_), 23.9 (C_6_H_10_), 23.5 (C_6_H_10_) ppm. ^31^P{^1^H} NMR (202.44 MHz, CDCl_3_, 25°C): δ = 132.7 ppm.

#### Dichlorido(η^6^-toluene)(3,5,6-bicyclophosphite-1,2-O-isopropylidene-α-D-glucofuranose)ruthenium(II) 3

The title compound was synthesized from 3,5,6-bicyclophosphite-1,2-*O*-isopropylidene-α-D-glucofuranose (99 mg, 0.4 mmol) and [(η^6^-toluene)RuCl(μ-Cl)]_2_ (106 mg, 0.2 mmol) following the general procedure.

Yield: 201 mg (97%); m.p. 180–181°C (decomp); elemental analysis calcd. for C_16_H_21_Cl_2_O_6_PRu·0.25CH_2_Cl_2_: C 36.58, H 4.06; found: C 36.93, H 4.22%; MS (ESI^+^): *m/z*: 534.6 [M + Na]^+^; ^1^H NMR (500.10 MHz, CDCl_3_, 25°C): δ = 6.19 [d, ^3^*J*(H,H) = 3 Hz, 1 H; H-1], 5.80–5.82 (m, 2 H; H-Ar), 5.58 (brs, 2 H; H-Ar), 5.52 (brs, 1 H; H-Ar), 5.09–5.12 (m, 1 H; H-5), 4.81 (brs, 1 H; H-3), 4.74 [d, ^3^*J*(H,H) = 2 Hz, 1 H; H-2], 4.44–4.48 (m, 1 H; H-6), 4.31–4.33 (m, 2 H; H-6′, H-4), 2.34 (s, 3 H; Ar-CH_3_), 1.52 [s, 3 H; C(CH_3_)_2_], 1.36 [s, 3 H; C(CH_3_)_2_] ppm. ^13^C{^1^H} NMR (125.75 MHz, CDCl_3_, 25°C): δ = 113.0 [*C*(CH_3_)_2_], 112.6 (C-Ar), 105.7 (C-1), 91.0 (C-Ar), 90.6 (C-Ar), 83.7 [^3^*J*(C,P) = 6 Hz, C-2], 82.2 (C-Ar), 81.8 (C-Ar), 79.2 [^3^*J*(C,P) = 8 Hz, C-3], 77.2 (C-Ar), 76.8 [^3^*J*(C,P) = 6 Hz, C-4], 74.8 [^3^*J*(C,P) = 5 Hz, C-5], 69.9 [^3^*J*(C,P) = 8 Hz, C-6], 26.9 [C(CH_3_)_2_], 26.3 [C(*C*H_3_)_2_], 19.2 (Ar-*C*H_3_) ppm. ^31^P{^1^H} NMR (202.44 MHz, CDCl_3_, 25°C): δ = 134.5 ppm.

#### Dichlorido(η^6^-toluene)(3,5,6-bicyclophosphite-1,2-O-cyclohexylidene-α-D-glucofuranose)ruthenium(II) 4

The title compound was synthesized from 3,5,6-bicyclophosphite-1,2-*O*-cyclohexylidene-α-D-glucofuranose (58 mg, 0.2 mmol) and [(η^6^-toluene)RuCl(μ-Cl)]_2_ (53 mg, 0.1 mmol) following the general procedure.

Yield: 107 mg (95%); m.p. 281–283°C (decomp); elemental analysis calcd. for C_19_H_25_Cl_2_O_6_PRu·0.15CH_2_Cl_2_: C 39.37, H 4.40; found: C 39.06, H 4.40%; MS (ESI^+^): *m/z*: 574.6 [M + Na]^+^; ^1^H NMR (500.10 MHz, CDCl_3_, 25°C): δ = 6.18 [d, ^3^*J*(H,H) = 4 Hz, 1 H; H-1], 5.80–5.82 (m, 2 H; H-Ar), 5.58 (brs, 2 H; H-Ar), 5.48–5.51 (m, 1 H; H-Ar), 5.08–5.12 (m, 1 H; H-5), 4.82 (brs, 1 H; H-3), 4.73 (brs, 1 H; H-2), 4.44–4.48 (m, 1 H; H-6), 4.32–4.34 (m, 2 H; H-6′, H-4), 2.34 (s, 3 H; Ar-CH_3_), 1.67–1.70 (m, 4 H; C_6_H_10_), 1.56–1.58 (m, 6 H; C_6_H_10_) ppm. ^13^C{^1^H} NMR (125.75 MHz, CDCl_3_, 25°C): δ = 113.7 (*C_cyc_*), 112.7 (C-Ar), 105.3 (C-1), 91.1 (C-Ar), 90.7 (C-Ar), 83.4 [^3^*J*(C,P) = 7 Hz, C-2], 82.3 (C-Ar), 81.6 (C-Ar), 79.4 [^3^*J*(C,P) = 8 Hz, C-3], 77.4 (C-Ar), 76.6 [^3^*J*(C,P) = 6 Hz, C-4], 74.9 [^3^*J*(C,P) = 5 Hz, C-5], 69.8 [^3^*J*(C,P) = 8 Hz, C-6], 36.9 (C_6_H_10_), 36.2 (C_6_H_10_), 24.8 (C_6_H_10_), 24.2 (C_6_H_10_), 23.5 (C_6_H_10_), 19.2 (*C*H_3_) ppm. ^31^P{^1^H} NMR (202.44 MHz, CDCl_3_, 25°C): δ = 134.4 ppm.

#### Dichlorido(η^6^-biphenyl)(3,5,6-bicyclophosphite-1,2-O-isopropylidene-α-D-glucofuranose)ruthenium(II) 5

The title compound was synthesized from 3,5,6-bicyclophosphite-1,2-*O*-isopropylidene-α-D-glucofuranose (99 mg, 0.4 mmol) and [(η^6^-biphenyl)RuCl(μ-Cl)]_2_ (131 mg, 0.2 mmol) following the general procedure.

Yield: 217 mg (91%); m.p. 178–179°C (decomp); elemental analysis calcd. for C_21_H_23_Cl_2_O_6_PRu·0.25CH_2_Cl_2_: C 42.85, H 3.98; found: C 42.97, H 3.88%; MS (ESI^+^): *m/z*: 596.6 [M + Na]^+^; ^1^H NMR (500.10 MHz, CDCl_3_, 25°C): δ = 7.69–7.71 (m, 2 H; H-Ar), 7.48–7.50 (m, 3 H; H-Ar), 6.18–6.22 (m, 3 H; H-Ar), 5.99 [d, ^3^*J*(H,H) = 4 Hz, 1 H; H-1], 5.88 (brs, 2 H; H-Ar), 5.03–5.07 (m, 1 H; H-5), 4.72 (brs, 1 H; H-3), 4.38–4.44 (m, 2 H; H-6′, H-2), 4.26 (brs, 1 H; H-4), 4.19 (brs, 1 H; H-6), 1.50 [s, 3 H; C(CH_3_)_2_], 1.33 [s, 3 H; C(CH_3_)_2_] ppm. ^13^C{^1^H} NMR (125.75 MHz, CDCl_3_, 25°C): δ = 133.7 (C-Ar), 130.1 (C-Ar), 129.0 (C-Ar), 128.8 (C-Ar), 128.7 (C-Ar), 127.3 (C-Ar), 112.5 [*C*(CH_3_)_2_], 108.3 (C-Ar), 105.7 (C-1), 91.2 (C-Ar), 90.8 (C-Ar), 89.9 (C-Ar), 88.9 (C-Ar), 88.2 (C-Ar), 83.5 [^3^*J*(C,P) = 5 Hz, C-2], 79.1 [^3^*J*(C,P) = 8 Hz, C-3], 76.8 (C-4), 74.9 [^3^*J*(C,P) = 5 Hz, C-5], 69.6 (C-6), 26.9 [C(*C*H_3_)_2_], 26.3 [C(*C*H_3_)_2_] ppm. ^31^P{^1^H} NMR (202.44 MHz, CDCl_3_, 25°C): δ = 132.3 ppm.

#### Dichlorido(η^6^-biphenyl)(3,5,6-bicyclophosphite-1,2-O-cyclohexylidene-α-D-glucofuranose)ruthenium(II) 6

The title compound was synthesized from 3,5,6-bicyclophosphite-1,2-*O*-cyclohexylidene-α-D-glucofuranose (58 mg, 0.2 mmol) and [(η^6^-biphenyl)RuCl(μ-Cl)]_2_ (65 mg, 0.1 mmol) following the general procedure.

Yield: 119 mg (93%); m.p. 170–172°C (decomp); elemental analysis calcd. for C_24_H_27_Cl_2_O_6_PRu·0.3CH_2_Cl_2_: C 45.61, H 4.35; found: C 45.27, H 4.38%; MS (ESI^+^): *m/z*: 636.7 [M + Na]^+^; ^1^H NMR (500.10 MHz, CDCl_3_, 25°C): δ = 7.62–7.69 (m, 2 H; H-Ar), 7.46–7.49 (m, 3 H; H-Ar), 6.17–6.20 (m, 3 H; H-Ar), 5.98 [d, ^3^*J*(H,H) = 4 Hz, 1 H; H-1], 5.87 (brs, 2 H; H-Ar), 5.03–5.05 (m, 1 H; H-5), 4.73 (brs, 1 H; H-3), 4.43–4.46 (m, 2 H; H-6′, H-2), 4.26 (brs, 1 H; H-4), 4.18 (brs, 1 H; H-6), 1.65–1.67 (m, 4 H; C_6_H_10_), 1.54–1.58 (m, 6 H; C_6_H_10_) ppm. ^13^C{^1^H} NMR (125.75 MHz, CDCl_3_, 25°C): δ = 133.6 (C-Ar), 130.1 (C-Ar), 129.0 (C-Ar), 128.8 (C-Ar), 128.6 (C-Ar), 127.2 (C-Ar), 113.2 (*C_cyc_*), 108.5 (C-Ar), 105.4 (C-1), 91.3 (C-Ar), 90.6 (C-Ar), 90.0 (C-Ar), 89.0 (C-Ar), 88.3 (C-Ar), 83.2 [^3^*J*(C,P) = 6 Hz, C-2], 79.3 [^3^*J*(C,P) = 8 Hz, C-3], 76.6 (C-4), 75.0 [^3^*J*(C,P) = 5 Hz, C-5], 70.0 (C-6), 36.5 (C_6_H_10_), 35.8 (C_6_H_10_), 24.8 (C_6_H_10_), 23.9 (C_6_H_10_), 23.5 (C_6_H_10_) ppm. ^31^P{^1^H} NMR (202.44 MHz, CDCl_3_, 25°C): δ = 132.4 ppm.

#### Dichlorido(η^6^-phenoxyethanol)(3,5,6-bicyclophosphite-1,2-O-isopropylidene-α-D-glucofuranoside)ruthenium(II) 7

3,5,6-Bicyclophosphite-1,2-*O*-isopropylidene-α-D-glucofurano-side (200 mg, 0.8 mmol) was added to a solution of {(η^6^-phenoxyethanol)RuCl(μ-Cl)}_2_ (250 mg, 0.4 mmol) in CH_2_Cl_2_ (30 mL). The reaction mixture was stirred for 12 h at room temperature. The insoluble residue was removed by filtration and diethyl ether was added to a red solution in order to precipitate the crude product. The pure product was obtained by purification on a silica gel column with CH_2_Cl_2_: MeOH (20: 1) as eluent. Yield: 268 mg (60%); m.p. 120–121°C (decomp); elemental analysis calcd. for C_17_H_23_O_8_RuPCl_2_: C 36.57, H 4.15; Found: C 36.96, H 4.35%; MS (ESI^+^) *m/z*: 522 [M - Cl]^+^; ^1^H NMR (400.13 MHz, D_2_O, 25°C): δ = 6.25 [d, ^3^*J*(H,H) = 3.5 Hz, 1 H; H-1], 6.20 (m, 2 H; H-Ar), 5.65 (m, 2 H; H-Ar), 5.30 (m, 1 H; H-Ar), 5.24 (m, 1 H; H-5), 4.98 (m, 1 H; H-3), 4.89 [d, ^3^*J*(H,H) = 3.5 Hz, 1 H; H-2], 4.86 [dd, ^2^*J*(H,H) = 11.8 Hz, ^3^*J*(H,P) = 9.9 Hz, 1H; H-6], 4.57 (m, 1 H; H-4), 4.32 (m, 1 H; H-6′), 4.3 [t, ^3^*J*(H,H) = 4.3 Hz, 2 H; O-CH_2_-CH_2_-OH], 3.94 [t, ^3^*J*(H,H) = 4.3 Hz, 2 H; O-CH_2_-CH_2_-OH], 1.51 (s, 3 H; CH_3_), 1.37 (s, 3 H; CH_3_) ppm.^13^C{^1^H} NMR (100.63 MHz, CDCl_3_, 25°C): δ = 145.3 (C-Ar), 112.6 [C(CH_3_)_2_], 105.8 (C-1), 94.3 (C-Ar), 93.7 (C-Ar), 83.6 [^3^*J*(C,P) = 6.1 Hz; C-2], 79.0 [^2^*J*(C,P) = 8.0 Hz; C-3], 77.2 (C-4), 74.7 (C-Ar or C-5), 73.8 (C-Ar or C-5), 72.8 (C-Ar, O-CH_2_), 69.0 (C-6), 60.8 (CH_2_-OH), 26.9 (CH_3_), 26.2 (CH_3_) ppm.^31^P{^1^H} NMR (161.98 MHz, CDCl_3_, 25°C): δ = 137.1 ppm.

#### Dichlorido(η^6^-phenoxyethanol)(3,5,6-bicyclophosphite-1,2-O-cyclohexylidene-α-D-glucofuranoside)ruthenium(II) 8

3,5,6-Bicyclophosphite-1,2-*O*-cyclohexylidene-α-D-glucofuranoside (230 mg, 0.8 mmol) was added to a solution of {(η^6^-phenoxyethanol)RuCl(μ-Cl)}_2_ (250 mg, 0.4 mmol) in CH_2_Cl_2_ (30 mL). The reaction mixture was stirred for 12 h at room temperature. The insoluble residue was removed by filtration and diethyl ether was added to a red solution in order to precipitate the crude product. The pure product was obtained by purification on a silica gel column with CH_2_Cl_2_: MeOH (20: 1) as eluent. Yield: 350 mg (73%); m.p. 90–91°C (decomp); elemental analysis calcd. for C_20_H_27_O_8_RuPCl_2_: C 40.14, H 4.55; Found: C 40.41, H 4.73%; MS (ESI^+^) *m/z*: 563 [M - Cl]^+^; ^1^H NMR (400.13 MHz, CD_2_Cl_2_, 25°C): δ = 6.19 [d, ^3^*J*(H,H) = 4 Hz, 1 H; H-1], 5.98 (m, 2 H; H-Ar), 5.53 (m, 2 H; H-Ar), 5.13 (m, 1 H; H-Ar), 5.10 (m, 1 H; H-5), 4.82 (m, 1 H; H-3), 4.73 [d, ^3^*J*(H,H) = 4 Hz, 1 H; H-2], 4.50 [dd, ^2^*J*(H,H) = 12 Hz, ^3^*J*(H,P) = 9 Hz, 1 H; H-6], 4.40 [t, ^3^*J*(H,H) = 4 Hz, 2H; O-CH_2_-CH_2_-OH], 4.33 (m, 1 H; H-4), 4.28 (m, 1 H; H-6′), 3.97 [t, ^3^*J*(H,H) = 4 Hz, 2H; O-CH_2_-CH_2_-OH], 3.37 (brs, 1 H;OH), 1.70–1.24 (m, 10 H; CH_2_) ppm. ^13^C{^1^H} NMR (100.63 MHz, CD_2_Cl_2_, 25°C): δ = 145.0 (C-Ar), 113.2 (C_6_H_10_), 105.4 (C-1), 94.0 (C-Ar), 93.6 (C-Ar), 83.3 [^3^*J*(C,P) = 6.0 Hz; C-2], 79.2 [^2^*J*(C,P) = 8.3 Hz; C-3], 76.8 [^3^*J*(C,P) = 4.9 Hz; C-4], 74.6 [^2^*J*(C,P) = 5.7 Hz; C-5], 73.7 (C-Ar), 72.9 (C-Ar), 72.7 (O-CH_2_), 69.2 [^2^*J*(C,P) = 9.2 Hz; C-6], 60.6 (CH_2_-OH), 36.4 (C_6_H_10_), 35.7 (C_6_H_10_), 24.8 (C_6_H_10_), 23.9 (C_6_H_10_), 23.6 (C_6_H_10_) ppm. ^31^P{^1^H} NMR (161.98 MHz, CD_2_Cl_2_, 25°C): δ = 137.1 ppm.

#### Hydrolysis and reactivity with 9-ethylguanine (9-ETG)

For hydrolysis studies, the compounds were dissolved in D_2_O and the samples were analyzed by ^1^H and ^31^P{^1^H} NMR spectroscopy after 1, 24, 48 and 96 h. For 9-ethylguanine binding experiments, the complexes were mixed at molar ratios of 1: 1 and 1: 2 (complex: 9-EtG) in D_2_O and reaction progress was monitored by ^1^H and ^31^P{^1^H} NMR spectroscopy after 1, 24 and 96 h, while samples were kept at room temperature during this time period.

#### DNA interaction studies

The structural modification of DNA by **2, 4**, and **6** was tested by agarose gel electrophoresis with the plasmid pBR322. TAE (1x) buffer was employed as incubation medium. Stock solutions of 1 mM of complex **2, 4**, and **6** were prepared in TAE (1x) buffer, diluted with TAE (1x) buffer and stored at −20 °C. The plasmid pBR322 was diluted with TAE (1x) buffer as well. Incubation mixtures were prepared to yield *r*_b_-values corresponding to 0.01, 0.05, 0.10, 0.50, 1.00 and 5.00.

The agarose gels consisted of 1% agarose in TAE (1×) buffer, and the incubation mixtures were subject to 35 min running time at 100 V and 70 mA in a PerfectBlue™ Mini S (PEQLAB) GE chamber. The DNA bands were stained with ethidium bromide (1 μl/mL) and were processed and analyzed with the gel documentation system GenoView UV-source and GenoSoft Version 3.08 C (VWR).

#### Cathepsin B inhibition assay

Crude bovine spleen cathepsin B (cat B) was purchased from Sigma (C6286) and used without further purification. The colorimetric cat B assay was performed in 100 mM sodium phosphate, 1 mM EDTA, 0.025% polyoxyethylene (23) lauryl ether (BRIJ), pH 6.0, using Na-CBZ-L-lysine *p*-nitrophenyl ester (CBZ = *N*-carbobenzoxy) as substrate. For the enzyme to be catalytically functional, the active site cysteine needs to be in a reduced form which was accomplished before dilution with an excess of dithiothreitol (DTT) for 1 h at 30°C. IC_50_ determinations were performed in triplicate using a fixed enzyme concentration of 500 nM and a fixed substrate concentration of 200 μM. Inhibitor concentrations ranged from 0.3 to 200 μM.

The enzyme and inhibitor were co-incubated at 30°C over a period of 24 h prior to the addition of substrate. Activity was measured over 3 min at 326 nm. Colorimetric readings were performed on a Lambda 20 Bio spectrophotometer (Perkin-Elmer).

#### Cytotoxicity studies

***Cell lines and culture conditions.*** CH1 cells originate from an ascites sample of a patient with a papillary cystadenocarcinoma of the ovary and were a gift from Lloyd R. Kelland, CRC Centre for Cancer Therapeutics, Institute of Cancer Research, Sutton, UK. SW480 (adenocarcinoma of the colon, human), and A549 (non-small cell lung cancer, human) cells were provided by Brigitte Marian (Institute of Cancer Research, Department of Medicine I, Medical University of Vienna, Austria). All cell culture media and reagents were obtained from Sigma-Aldrich Austria. Cells were grown in 75 cm^2^ culture flasks (Iwaki) as adherent monolayer cultures in Eagle's Minimal Essential Medium (MEM) supplemented with 10% heat-inactivated fetal calf serum, 1% v/v non-essential amino acids (from 100× ready-to-use stock), 1 mm sodium pyruvate and 4 mm L-glutamine (complete medium). Cultures were maintained at 37°C in a humidified atmosphere containing 95% air and 5% CO_2_.

***MTT assay conditions.*** Cytotoxicity was determined using the colorimetric MTT (3-(4,5-dimethyl-2-thiazolyl)-2,5-diphenyl-2*H*-tetrazolium bromide, Fluka) assay. CH1, SW480 and A549 cells were harvested from the culture flasks by trypsinization, and 100 μL aliquots were seeded into 96-well microculture plates (Iwaki/Asahi Technoglass, Gyouda, Japan) in complete medium. Densities of 1.5 × 10^3^ (CH1), 2.5 × 10^3^ (SW480) and 4 × 10^3^ (A549) cells per well were chosen in order to ensure exponential growth of untreated controls throughout the experiment. Cells were allowed to settle for 24 h. Then, the test compounds were dissolved and serially diluted in complete medium, and 100 μL aliquots were added to the microcultures. Cells were exposed to the test compounds for 96 h. After exposure, all media were replaced with 1:6 MTT/RPMI mixture (100 μL per well) containing MTT solution in phosphate-buffered saline (5 mg/ml) and MTT/RPMI1640 culture medium (supplemented with 10% heat-inactivated fetal calf serum and 4 mM L-glutamine). After incubation for 4 h, the supernatants were removed, and the formazan crystals formed in viable cells were dissolved in 150 μL DMSO per well. Optical densities were measured at 550 nm with a microplate reader (Tecan Spectra Classic), using a reference wavelength of 690 nm to correct the unspecific absorption. The quantity of viable cells was expressed in terms of T/C values by comparison to untreated control, and 50% inhibitory concentrations (IC_50_) were calculated from concentration-effect curves by interpolation. Evaluation is based on means from at least three independent experiments, each comprising three replicates per concentration level.

#### Determination of cellular accumulation of the complexes

The cellular accumulation study was conducted following a previously published protocol (Egger et al., 2009). SW480 cells were seeded in 6-well plates (Iwaki/Asahi Technoglass, Gyouda, Japan) in 2.5 mL complete medium (MEM) per well in densities of about 3 × 10^5^ cells per well. Cell microcultures were incubated in a moist atmosphere at 37°C for 24 h prior to exposure to the test compounds. The cell number was determined using trypan blue staining in parallel microcultures during the 2 h exposure period. After exposure the medium was removed, cells were washed with PBS and consecutively lyzed with 0.5 mL sub-boiled HNO_3_ (conc.) per well for 1 h. The Ru concentration was quantified by ICP-MS in presence of 0.5 μM indium used as internal standard. Results are based on three independent experiments, each consisting of three replicates.

## Results and discussion

**Figure 2:**
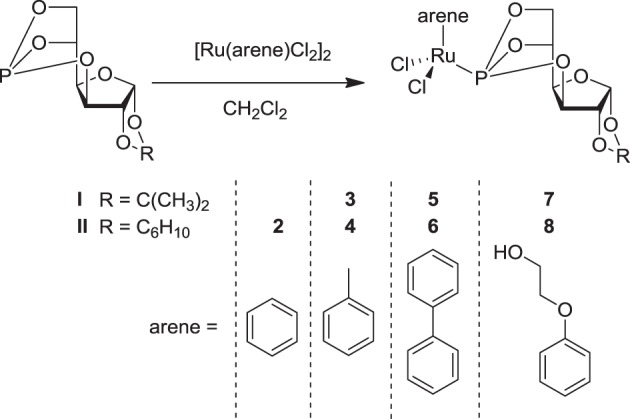
**Synthesis of the dichlorido–Ru^II^ compounds (2–8) with *P*-derived sugar ligands (I, II)**.

In order to further improve the specificity and efficacy of sugar-based RAPTA analogues, we have prepared further compounds carrying benzene, toluene, biphenyl and phenoxyethanol as the π-bound arene ligands, in order to explore the influence of the arene ligand on the antitumor activity of the compounds as well as on their modes of action. The organometallic Ru^II^-chlorido complexes **2–8** were synthesized by reacting 3,5,6-bicyclophosphite-1,2-*O*-isopropylidene-α-D-glucofuranoside **I** or 3,5,6-bicyclophosphite-1,2-*O*-cyclohexylidene-α-D-glucofuranoside **II** with the respective bis[dichlorido(η ^6^-arene)ruthenium(II)] precursor in CH_2_Cl_2_ (Figure 2) (Berger et al., 2008; Hanif et al., 2010a). All the complexes were obtained in very good yield and were characterized by 1D and 2D NMR spectroscopy, ESI-MS, and elemental analysis. In the ^1^H NMR spectra, protons of the coordinated arene ligands give rise to signals that appear in the range 5–6 ppm. Complexes **5** and **6**, which contain a η^6^-biphenyl ligand, exhibit additional resonances due to the aromatic protons from the second phenyl ring at *ca*. 7–8 ppm. The benzene ligand in **2** gives a singlet resonance at 6.20 ppm. The coordination of the P-donor sugar-based ligands to the metal center results in a dramatic change in the ^31^P{^1^H} NMR singlet resonance from *ca*. 117 to 135 ppm, which is similar to that of analogous compounds (Berger et al., 2008). Moreover, the presence of the NMR active phosphorus nucleus induces splitting of some of the carbon signals of coordinated arene moieties due to P-C coupling (Berger et al., 2008). ESI-MS further confirms the structures with peaks of high relative intensity that may be assigned to [M+Na]^+^ or [M–Cl]^+^ ions for all compounds.

The hydrolysis of **2, 3, 7**, and **8** was studied by ^31^P{^1^H} NMR spectroscopy and parallels earlier findings for 1a (Berger et al., 2008). Dissolution of **2** and **3** in water results after 1 h-incubation in a single peak at about 136 ppm, which is assignable to the parent compound and indicates stability within this time period. Within 24 h, two additional peaks of equal relative intensity at 137.4 and 136.8 ppm appear due to formation of diastereomers by exchange of a single chlorido ligand with an aqua molecule. This was accompanied by the cleavage of a P–O bond as indicated by signals of equal relative intensity at 95.7 and 94.5 ppm, while an additional signal at 94.7 ppm is assigned to a compound in which P–O bond cleavage occurred. The ^31^P{^1^H} NMR spectrum recorded after 48 h contains two additional signals at 123.8 and 122.4 ppm, probably due to formation of dimeric species. The sequence of formation of hydrolytic species is similar to that observed for related ruthenium(II) compounds (Scolaro et al., 2005; Berger et al., 2008; Scolaro et al., 2008; Hanif et al., 2010a; Nazarov et al., 2012). Extending the reaction time to 96 h did not result in the formation of additional species, and it seems that an equilibrium has been reached with 60–70% of the compound remaining in its original form. A similar process was observed for **7** and **8**, demonstrating that the addition of a hydroxyl functionality to the arene ligand does not alter the aquation profile.

### Biological evaluation

**Figure 3:**
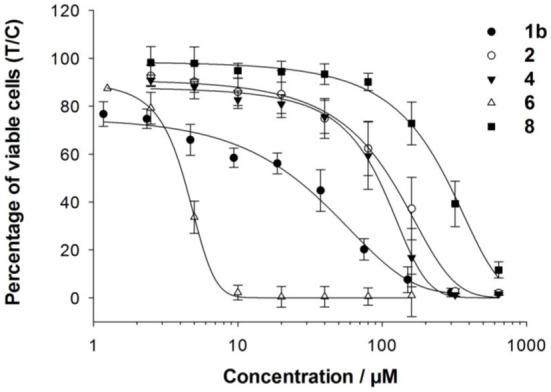
**Concentration–effect curves of 1b, 2, 4, 6, and 8 in CH1 cells**.

The cancer-inhibiting potential of **2–6** and **8** was determined in human SW480 colon adenocarcinoma, CH1 ovarian cancer and A549 non-small cell lung cancer cells by using the MTT assay. The obtained results are compared to those of analogous Ru(II) compounds in Table 1, and the concentration–effect curves of **1b, 2, 4, 6**, and **8** in CH1 cells, which are usually most sensitive to the treatment with metal compounds, are shown in Figure 3. Variation in the arene ligand of the Ru complexes has a strong influence on antiproliferative activity, which is improved with increasing the lipophilic character of the arene and carbohydrate ligands in line with the cellular accumulation of the compounds (Table 1).

**Table 1:** **Cellular accumulation in SW480 colon cancer cells and *in vitro* anticancer activity (mean IC_50_ values ± standard deviations) of 1–6 and 8 in human ovarian cancer (CH1), colon adenocarcinoma (SW480), and non-small cell lung cancer (A549) cells (exposure time 96 h)**.

**Compound**	**Cellular uptake/Ru [fg/cell]**	**IC_50_ values/μM**		
		**CH1**	**SW480**	**A549**
**1a**	10 ± 2	60 ± 14^a^	361 ± 122^a^	498 ± 17^a^
**1b**	20 ± 2	29 ± 4^a^	150 ± 19^a^	223 ± 14^a^
**2**	29 ± 6	118 ± 36	129 ± 13	417 ± 68
**3**	n.d.	164 ± 47	276 ± 6	427 ± 163
**4**	18 ± 3	92 ± 22	99 ± 13	486 ± 55
**5**	74 ± 9	29 ± 3	26 ± 5	>160
**6**	224 ± 33	3.9 ± 0.3	5.3 ± 0.9	56 ± 9
**8**	4 ± 1	189 ± 3	260 ± 52	314 ± 32
RAPTA-C	n.d.	65 ± 15	171 ± 59	>515

a From Berger et al. (2008); n.d., not determined.

Carbohydrate compounds potentially accumulate in tumors due to their high demand for glucose as a result of their upregulated glycolytic energy production. Recently, fluorescent Ru(arene) complexes bearing the pta or a sugar phosphite ligand were shown to accumulate in cells to a similar extent (Nazarov et al., 2012). In the present study, the most lipophilic compound 6, according to aqueous solubility, with the biphenyl ligand and a cyclohexylidene moiety at the sugar-derived ligand was most efficiently taken up into the cell and was also the most active derivative with IC_50_ values in the low micromolar range. The following order of cytotoxicity was observed with regard to variation of the arene ligand in **1b, 2, 4** and **6** with the identical sugar moiety: biphenyl > cymene > toluene > benzene.

Complex **3** was chosen for DNA interaction studies with 9-ethylguanine (9-EtG), and the reaction was monitored by NMR spectroscopy. Complex **3** appears to form adducts with the DNA model nucleobase 9-EtG via *N*7 of the guanine moiety as shown by ^1^H NMR spectroscopy where a low-field shift from 7.85 to 8.29 ppm was observed for H8 of guanine. Analysis by ^31^P{^1^H} NMR spectroscopy immediately after mixing **3** with 9-EtG revealed a single peak at 135.9 ppm for the unreacted compound. Within 1 h, two additional signals at 136.6 and 137.2 ppm appear due to the formation of diastereomers by exchange of one of the chlorido ligands with 9-EtG. However, after 24 h several species were formed, including signals at 93.3 and 95.1 ppm indicative of cleavage of a P-O bond, similar to the behavior observed for related analogous complexes (Berger et al., 2008).

Furthermore, the reactivity to DNA and impact on its secondary structure was studied by determining the electrophoretic mobility of a dsDNA plasmid in cell-free experiments. However, no influence of complexes **2, 4**, or **6** on the electrophoretic mobility of pBR322 plasmid DNA was observed at r_b_-values (metal-to-nucleobase ratio) up to 5 within the incubation period of 2 h. Consequently, the incubation period was increased to 4 and 6 h for complexes **4** and **6**, which did not lead to any observable change either, making it unlikely that the cytotoxic effect is related to DNA binding interactions.

For this reason the compounds were evaluated as inhibitors of cat B since bifunctional ruthenium(arene) compounds have been shown to be good inhibitors (Casini et al., 2008). Cathepsin B has been proposed to participate in metastasis, angiogenesis, and tumor progression, and therefore it is believed to be an important target for the control of tumor progression (Mohamed and Sloane, 2006). Cat B inhibitors reduce both tumor cell motility and invasiveness *in vitro* (Frlan and Gobec, 2006), and many palladium, rhenium, gold and ruthenium complexes, such as members of the RAPTA family and particularly RAPTA-C and RAPTA-T (Casini et al., 2008), inhibit cat B potently (Casini et al., 2008; Mura et al., 2010). Indeed, the sugar-derived phosphite based ruthenium complexes **1a, 1b**, and **6** exhibit IC_50_ values in the low micromolar range (Table 2) and thus inhibit cat B to an extent similar to RAPTA-T. However, no correlation with cytotoxicity data was observed.

**Table 2:** **IC_50_ (μM) of 1a, 1b, RAPTA-C, and RAPTA-T against bovine cat B**.

**Compound**	**IC_50_ values/μM Cat B**
**1a**	1.5 ± 0.2
**1b**	4.0 ± 0.6
**6**	8.0 ± 0.4
RAPTA-C	2.5 ± 0.5 ^a^
RAPTA-T	1.5 ± 0.2 ^a^

a From Casini et al. (2008).

## Concluding remarks

In this paper, we report on the synthesis and biological evaluation of Ru^II^(η^6^-arene) complexes with carbohydrate-derived phosphorus-containing ligands. The cytotoxicity appears to be dependent on the lipophilicity, i.e., the most lipophilic compound is the most cytotoxic. Moreover, such complexes are known to exhibit certain selectivity for tumor cells over non-tumorigenic cells and are also active in cisplatin-resistant cancer cells (Berger et al., 2008; Hanif et al., 2010a). The arene ligand has little influence on the hydrolysis behavior. Binding to 9-EtG as assayed by NMR spectroscopy suggests that some covalent interaction with DNA is possible, but no effect on the electrophoretic mobility and therewith the secondary structure of plasmid DNA was observed. Studies on the inhibition of cathepsin B revealed that the carbohydrate compounds are potent inhibitors comparable to the metastasis inhibitors RAPTA-C and RAPTA-T, which appear to interact with histone proteins of the nucleosome core particle (Wu et al., 2011). However, further experiments are required to draw unambiguous conclusions on the mode of action of these compounds. Overall, carbohydrate-based metal(arene) complexes have demonstrated promising antiproliferative activity in *in vitro* assays, but only *in vivo* experiments will give deeper insight into the potential of these compounds as anticancer drugs.

### Conflict of interest statement

The authors declare that the research was conducted in the absence of any commercial or financial relationships that could be construed as a potential conflict of interest.
